# Supplementary material for: Neuroprotective Effects of VEGF-B in a Murine Model of Aggressive Neuronal Loss with Childhood Onset
Source: Int J Mol Sci. 2025 Jan 10;26(2):538. doi: 10.3390/ijms26020538 (PMC11765331; doi:10.3390/ijms26020538)
Supplement: Supplementary file 1 [file ijms-26-00538-s001.zip › Supplementary figures text.pdf]

Figure S1. Expression of the IGF1R and VEGFR1 in the cerebellum. (A, B) Micrographs showing the expression of IGF1R (yellow) in Purkinje cells (Cb28k, cian) of WT (A) and PCD (B) mice at P25. (C, D) Micrographs of Purkinje cells labeled with VEGFR1 (yellow) and calbindin (Cb28k, cian) of WT (C) and PCD (D) mice at P15. Note that both receptors can be observed in the three cerebellar layers. Scale bar 10 μm

Figure S2. Heat maps of the animal's movement during the Novel object recognition and three-chamber social preference test in PCD-rhIGF1 and PCD-VEGFB at P30. These maps show where the animal spent the most time, with the option to select between two categories: Familiar (Fam) and Novel (Nv) objects in the novel object recognition test, or between an object (Obj) and a mouse (Mouse) in the three-chamber social preference test.

Figure S3. Purkinje cell appearance. Images of single neurons from WT (A), PCD (B), PCD-IGF1 (C) and PCD-VEGFB (D) mice. Note that the Purkinje cell morphology (labeled with calbindin Cb28k, red) observed in both WT and PCD-VEGFB animals is qualitatively similar, exhibiting the characteristic and wide arborizing dendritic tree. However, there are noticeable morphological alterations in both PCD and PCD-rhIGF1 samples, especially in the dendritic tree. Scale bar 20 μm.

Figure S4. Effect of rhVEGF-B treatment on programmed cell death at P25. Caspase 3 labelling (cyan) in sagittal slices of cerebellum from WT (A), PCD (B) and PCD-VEGFB (C) mice. Purkinje cells are labeled with calbindin Cb28k (red). Note the increase in apoptosis in PCD and PCD-rhVEGFB compared to WT animals. Scale bar 200 μm.

Figure S5. Effect of rhVEGF-B treatment on skeletal muscle at P30. (A-C) Micrographs of longitudinally sectioned skeletal muscle from WT mice (A) as an example of the measurement of the maximum (B) and minimum (C) muscle fiber length (dashed lines). (D, E) Estimation of the major (D) and minor (E) axis mean lengths of skeletal muscle in longitudinal section. (F, G) Micrographs of skeletal muscle from a WT mouse in cross section. (H) Quantification of the mean area of muscle fibers cut transversely. No differences were detected between experimental groups at any sectioning position. Scale bar 50 μm.

Figure S6. Experimental design of the different treatments evaluated in this study, the main analyses carried out and their relationship with the neurodegenerative process of the PCD mouse. (A) Timeline details of rhIGF-1 administration, the time points for behavioral tests and tissue collection for Purkinje cell survival analyses. (B) Timeline details of rhVEGF-B administration, the timepoints for behavioral tests and tissue collection for Purkinje cell survival, apoptotic and muscular analyses.
