# Supplementary figures and images for: Neuroprotective Effects of VEGF-B in a Murine Model of Aggressive Neuronal Loss with Childhood Onset
Source: Int J Mol Sci. 2025 Jan 10;26(2):538. doi: 10.3390/ijms26020538 (PMC11765331; doi:10.3390/ijms26020538)

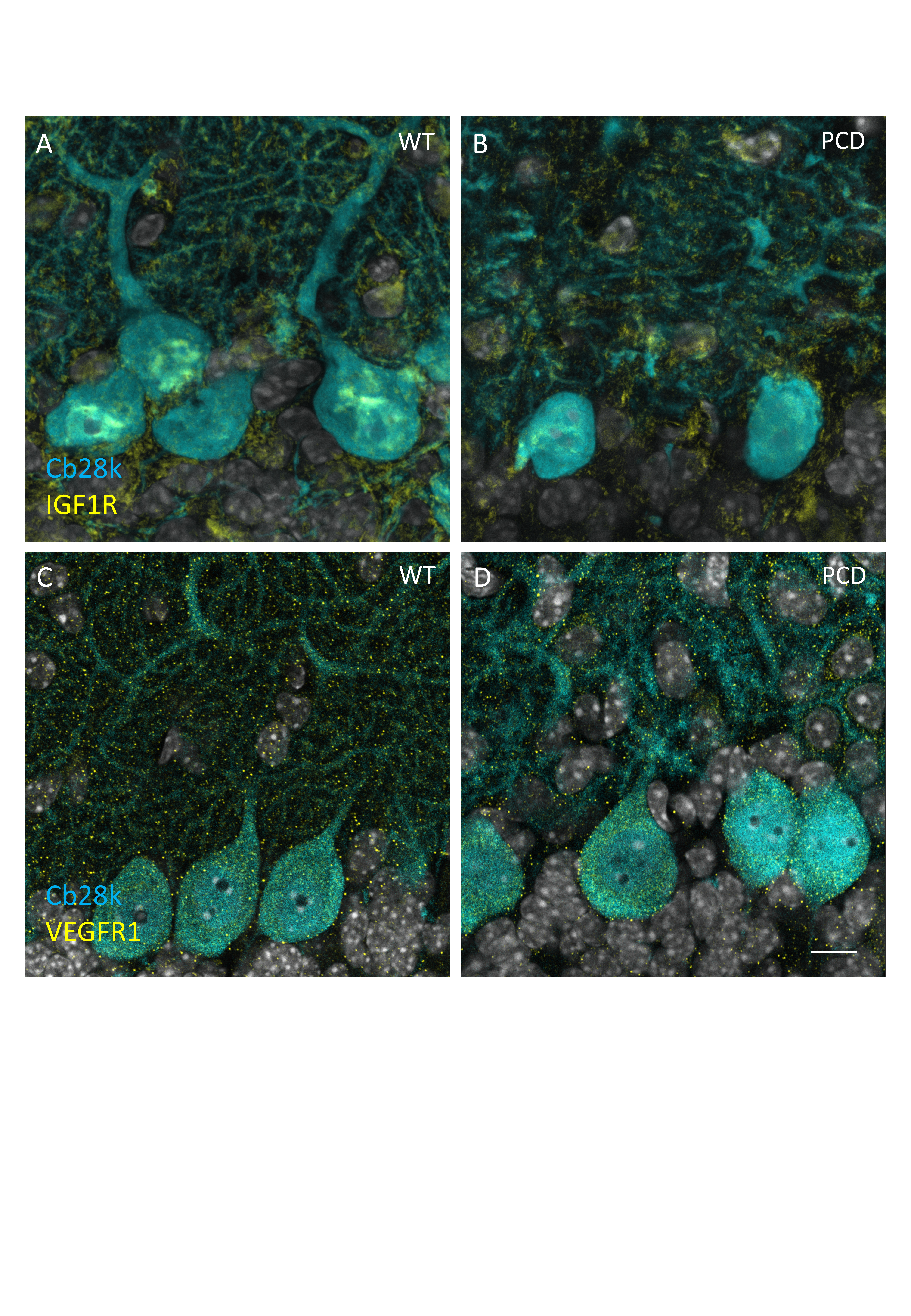

Supplement: Supplementary file 1 [file ijms-26-00538-s001.zip › Figure S1.tif]

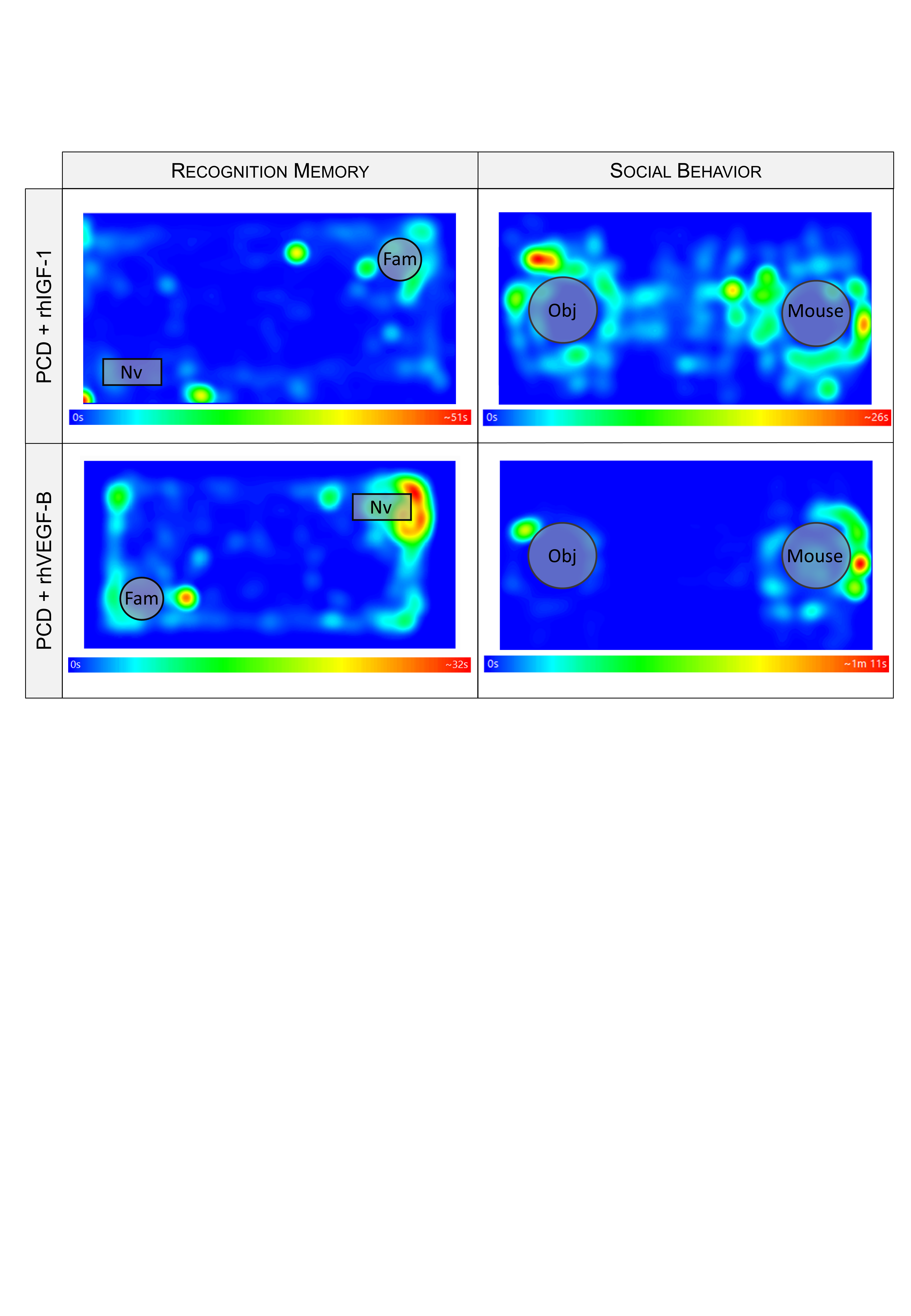

Supplement: Supplementary file 1 [file ijms-26-00538-s001.zip › Figure S2.tif]

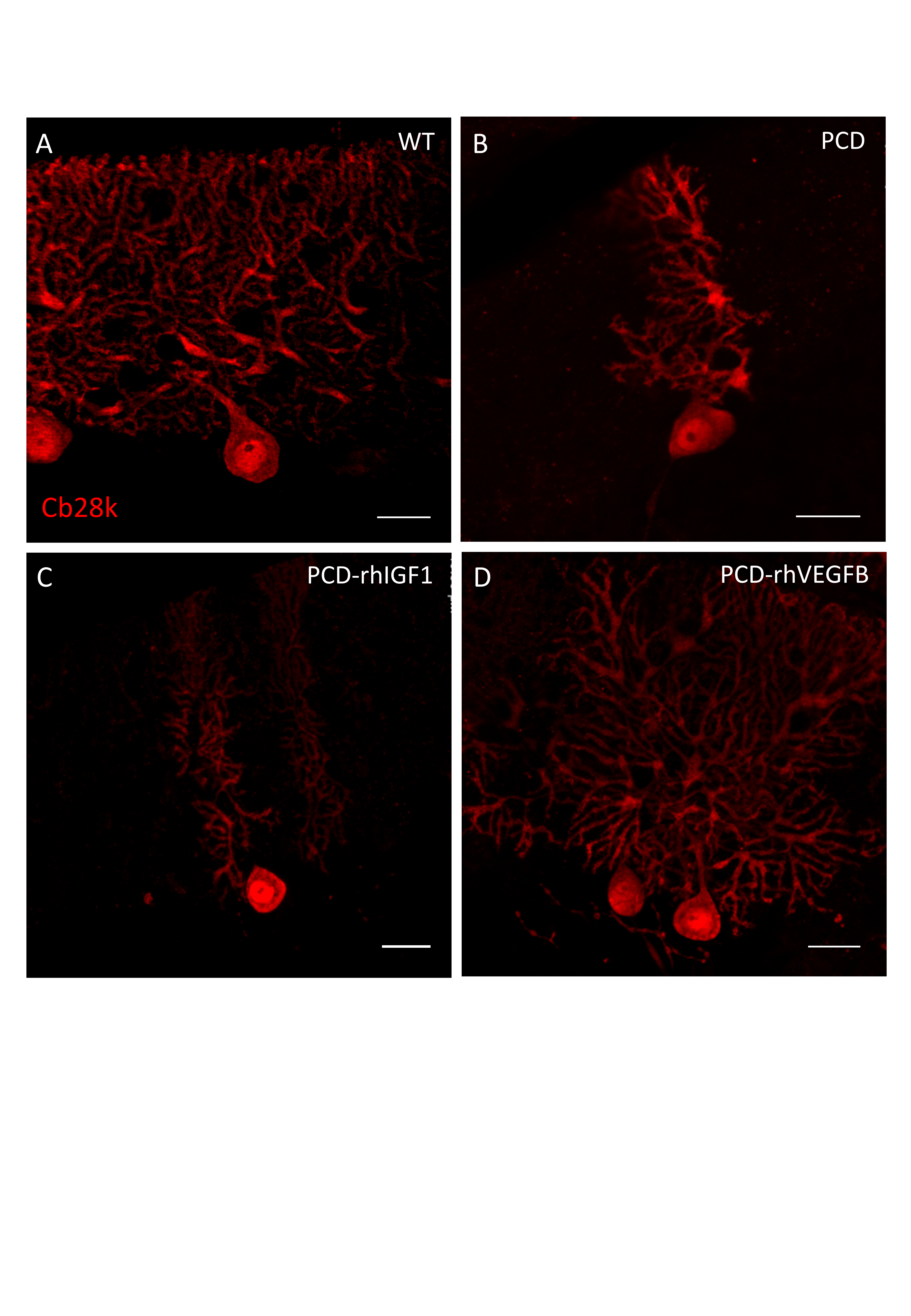

Supplement: Supplementary file 1 [file ijms-26-00538-s001.zip › Figure S3.tif]

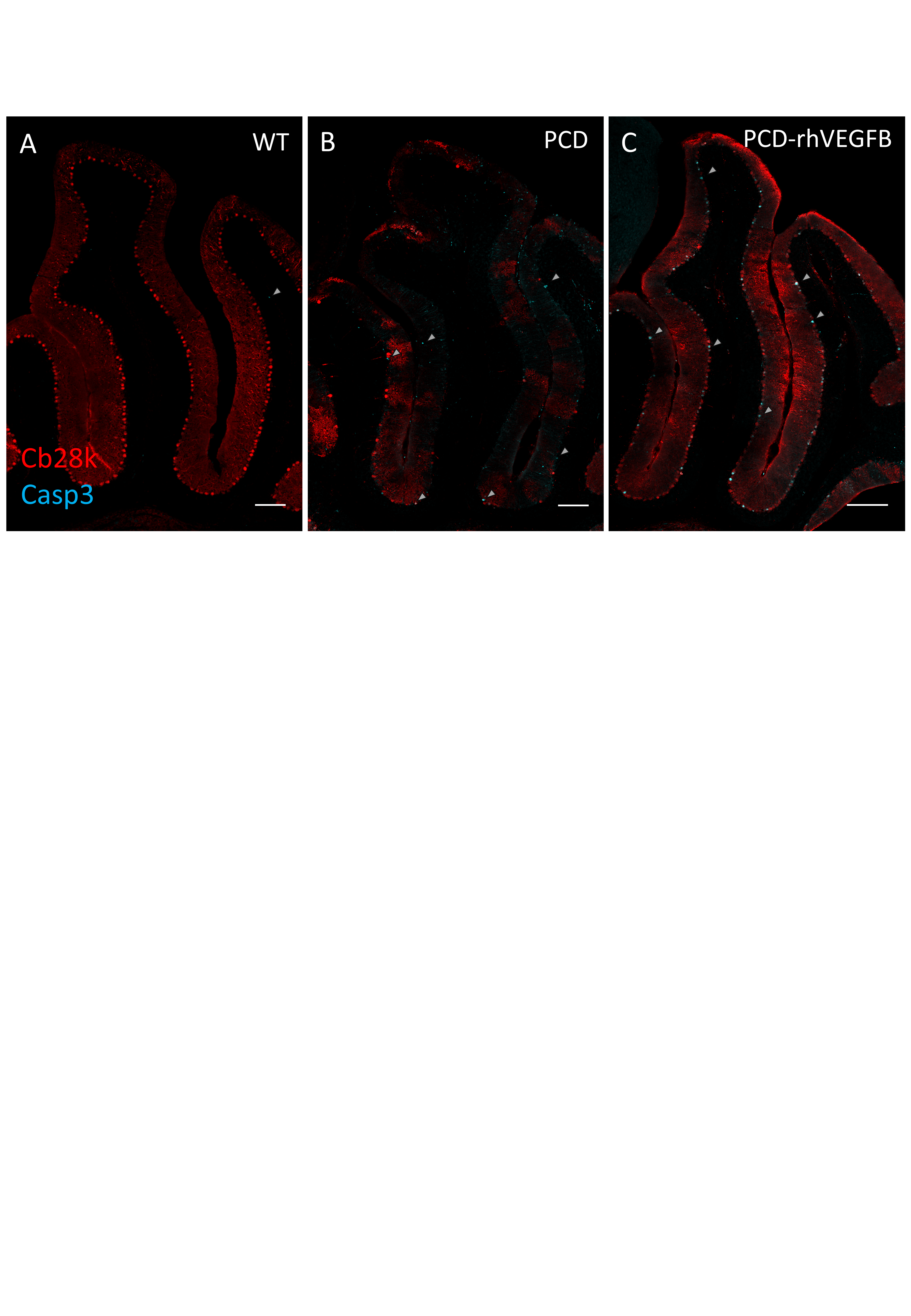

Supplement: Supplementary file 1 [file ijms-26-00538-s001.zip › Figure S4.tif]

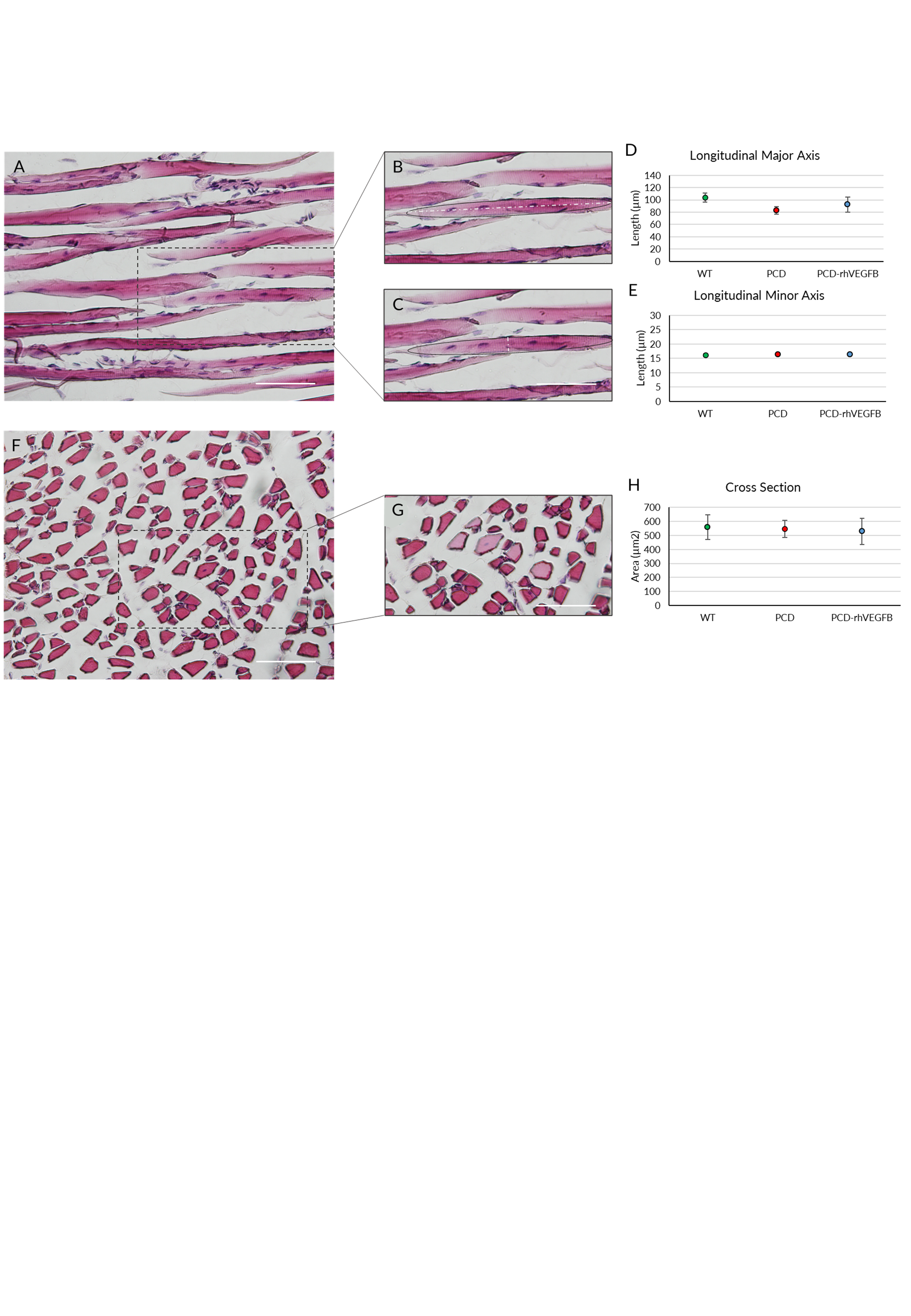

Supplement: Supplementary file 1 [file ijms-26-00538-s001.zip › Figure S5.tif]

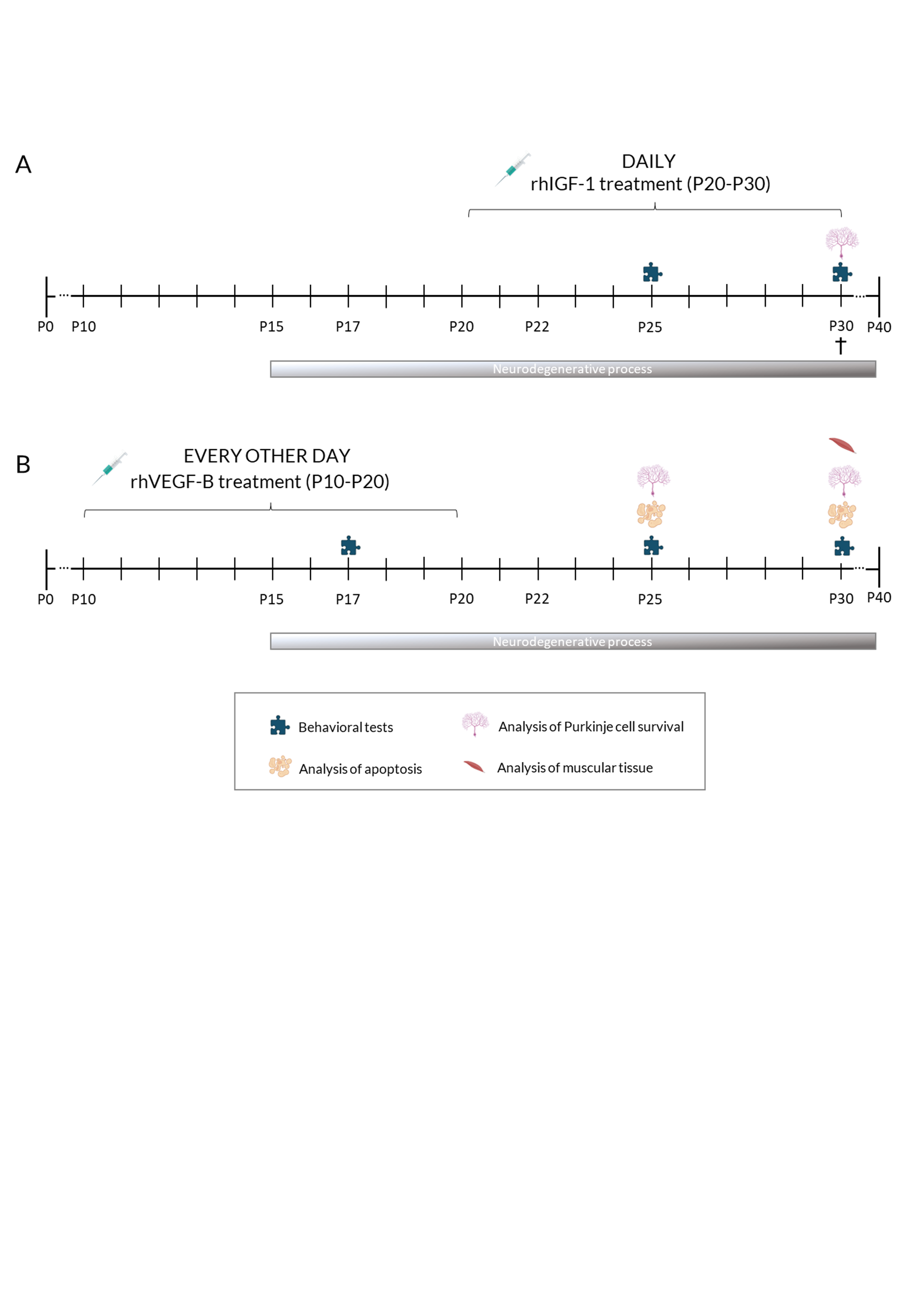

Supplement: Supplementary file 1 [file ijms-26-00538-s001.zip › Figure S6.tif]
